# Risk behaviour determinants among people who inject drugs in Stockholm, Sweden over a 10-year period, from 2002 to 2012

**DOI:** 10.1186/s12954-017-0184-8

**Published:** 2017-08-16

**Authors:** Niklas Karlsson, Michele Santacatterina, Kerstin Käll, Maria Hägerstrand, Susanne Wallin, Torsten Berglund, Anna Mia Ekström

**Affiliations:** 1 Department of Public Health Sciences, Karolinska Institutet, Stockholm, Sweden; 2 Department of Monitoring and Evaluation, Public Health Agency of Sweden, 171 82 Solna, Sweden; 3 Unit of Biostatistics, Institute of Environmental Medicine, Karolinska Institutet, Stockholm, Sweden; 4 Dependency Clinic, Linköping University Hospital, Linköping, Sweden; 5 Swedish Prison and Probation Service, Norrköping, Sweden; 6 Department of Infectious Diseases, Karolinska University Hospital, Stockholm, Sweden

**Keywords:** Determinants, HIV, Hepatitis C, Injection risk behaviour, People who inject drugs

## Abstract

**Background:**

People who inject drugs (PWID) frequently engage in injection risk behaviours exposing them to blood-borne infections. Understanding the underlying causes that drive various types and levels of risk behaviours is important to better target preventive interventions.

**Methods:**

A total of 2150 PWID in Swedish remand prisons were interviewed between 2002 and 2012. Questions on socio-demographic and drug-related variables were asked in relation to the following outcomes: Having shared injection drug solution and having lent out or having received already used drug injection equipment within a 12 month recall period.

**Results:**

Women shared solutions more than men (odds ratio (OR) 1.51, 95% confidence interval (CI) 1.03; 2.21). Those who had begun to inject drugs before age 17 had a higher risk (OR 1.43, 95% CI 0.99; 2.08) of having received used equipment compared to 17–19 year olds. Amphetamine-injectors shared solutions more than those injecting heroin (OR 2.43, 95% CI 1.64; 3.62). A housing contract lowered the risk of unsafe injection by 37–59% compared to being homeless.

**Conclusions:**

Women, early drug debut, amphetamine users and homeless people had a significantly higher level of injection risk behaviour and need special attention and tailored prevention to successfully combat hepatitis C and HIV transmission among PWID.

**Trial registration:**

ClinicalTrials.gov Identifier, NCT02234167

## Background

The level of coverage and uptake of needle exchange programmes (NEPs), antiretroviral therapy (ART), direct-acting antivirals (DAA) and opioid substitution treatment (OST) required to prevent hepatitis C (HCV) and HIV infections among people who inject drugs (PWID) is still under debate. With today’s effective medication against both HIV and HCV, it is plausible that a combination between treatment as prevention and other harm reduction services is needed to eliminate new infections among PWID [1, 2]. However, despite two decades of research on the association between injection risk behaviours and these blood-borne infections most often leaning more towards medical treatment alternatives [3, 4], the spread of HCV and HIV among PWID continues with significant public health, human and social costs [5]. Today, the research community is called upon to help policymakers prioritise and target existing primary and secondary prevention and harm reduction programmes to PWID most at risk [6], in particular, following the recent European Centre for Disease Prevention and Control (ECDC) report of an increase in the number of new HIV cases among PWID in several countries in Europe [7]. Better understanding of the mechanisms that influence or drive risk taking among PWID is therefore important.

Since the beginning of HIV and HCV surveillance in Sweden in 1983 and 1990, respectively, 11% of all new HIV infections and 40–65% of all registered HCV infections have been estimated to be associated with unsafe drug injection [8, 9]. NEPs and OST programmes have significantly reduced new HCV and HIV infections among PWID in many countries [1, 2], but Sweden has been very slow to introduce NEPs for political reasons [10]. Between 1986 and 2010, only two single NEP sites were active in the entire country and both were located in the most southern region, Skåne County, where also Sweden’s third NEP opened after 24 years. The capital city of Stockholm, which has the largest estimated number of PWID in Sweden [11] and a history of HIV outbreaks in this key population [12], opened its first NEP only a few years ago, in 2013 [8]. As of 2017, a total of 11 NEPs have opened in Sweden. With the recent paradigm shift in HCV treatment and in combination with NEP and OST programmes, the prospects of reducing the incidence of HCV among PWID are more promising than ever.

Sweden has among the lowest prevalence rates in Europe, with an estimated 0.36–0.41% and 0.07% of the Swedish population living with a known HCV or HIV infection, respectively [8]. The total number of newly registered HIV infections among PWID remains low with fewer than 10 cases reported annually during the last 5 years [8]. This is in contrast to the total number of newly registered HCV infections in Sweden, which remains high with approximately 900 cases reported annually in a total population of 10 million Swedes [8]. It is believed that approximately 65% of registered cases have been infected through drug-related injections [9]. The fact that treatment for HCV still is prohibitively expensive for providers and governments, even in high-income countries such as Sweden, severely restricts access to these life-saving drugs [13, 14] and reduces the treatment as prevention effect. A study in Stockholm found that more than 50% of PWID had HCV antibodies within 2 years after beginning to inject drugs [15], which consistently has been reported to take place around a median age of 19 years [16]. HCV transmission seems to continue among young PWID in Sweden with no visible reduction in incidence [8].

Injection risk behaviour has been demonstrated to decrease among PWID who are enrolled in a NEP [17]. In Sweden, the law has prohibited people below 20 years of age to access NEPs, making it very difficult to reach young PWID with these prevention efforts. Existing NEPs report up to 64% prevalence of HCV antibodies among PWID newly enrolled in such programmes in Sweden [18]. Continued scale-up of harm reduction services, such as NEP, OST programmes and an increased HCV and HIV treatment coverage among PWID, would most likely have a strong impact on the incidence of HCV and HIV [1, 2]; however, a majority contract HCV at a young age, before they are eligible to access NEPs [15, 19]. Therefore, we need a better understanding of the underlying reasons for sustained injection risk behaviour resulting in HCV and HIV transmission, to improve prevention efforts especially among young and most-at-risk PWID.

The historical lack of a systematic approach for PWID in Sweden has made it difficult to build a comprehensive understanding of the group in terms of size, their socio-demographic characteristics and injection risk behaviours except for those PWID who appear in the general health care system due to bacterial infections or injuries related to their lifestyle. The only platform for broader surveillance and to collect data over time is the Swedish Prison and Probation Service remand prisons. Remand prisons serve as temporary holding points for people in custody suspected but not charged of a criminal act, taking the form of open cohort nodes suitable for sentinel surveillance. Using a decade of remand prison surveillance data, we aimed to understand the underlying determinants for injection risk behaviour among PWID in Stockholm, Sweden.

## Methods

Each detained person entering into a Swedish remand prison is asked about general drug use [20]. To specifically target PWID, a project called ‘The Social Remand Prison Project’ was established in 2002 [21]. On a daily basis, social workers and nurses who were specifically trained and employed for the purpose of this project, completely separate from the remand prison, were given a list of newly arrived custodies to visit. The project staff, trained in interviewing techniques and injection drug knowledge, systematically visited people in their cells starting from the top of the list, which was sorted based on the time of arrival. During the visit, the detainee was offered an entirely voluntary basic health check-up including a test for HIV, HCV and hepatitis B (HBV). They were also asked, and could voluntarily answer, if they had ever used drugs and especially if they had injected any drugs. If the answer was yes, they were invited to participate in the project and to answer more questions about injection drug use. The project focus, i.e. to better understand determinants, risk behaviours and infectious diseases among PWID, was carefully explained verbally and in writing, and it was emphasised that participation was entirely voluntary and anonymous. Potential interviewees were also informed that project participation would not influence the outcome of the correctional process and that the respondent could choose to end the interview at any time without any negative consequences. The participants were thereafter asked to give informed verbal consent before the interview began. Thereafter an extended face-face interview took place performed by trained project staff as described above. All names on the questionnaire were replaced by coded study identification numbers that were unrelated to any personal identifiers. The questionnaire that had been carefully piloted included up to 80 questions on background factors, risk behaviours and infectious diseases [21].

### Inclusion criteria

We used surveillance data collected between 2002 and 2012 through face-to-face interviews as described above. A total of 3824 individuals who acknowledged that they were using some type of drug were identified as potentially eligible for inclusion in the current analysis. We then proceeded to select PWID, here defined as respondents with a non-missing or positive (yes) response to at least one of the following 12 questions: age when starting injection drugs, injection drug as the first drug used, the use of an injection drug during the last 12 months, the use of new injection equipment at their last injection (yes/no), having been provided free injection equipment during the last 12 months (yes/no), reporting an injection drug as the main drug used during the last 12 months, injection drug as the main drug of use, number of times the last injection needle was re-used, number of people with whom injection equipment were shared with during the last months, the sharing of injection drug solution during the last 12 months (i.e. filters, rinse water and drug mixtures, yes/no), and the lending out or receipt of already used injection equipment (i.e. needles or syringes, yes/no) from somebody during the last 12 months. All questions were asked relating to the time period/situation preceding the current arrest. A total of 1484 were excluded because they were defined as non-injection drug users. The focus of our study was to establish baseline knowledge among first-time detainees, which is why we only selected PWID at their first appearance in the Remand Prison Project for each year. If an individual was found to reappear later the same year, or later in the study period, he or she was excluded from re-appearing more than once in the data analysis and only data from the first visit in the Remand Prison Project was included (*N* = 136). We also excluded those who had only injected anabolic steroids (54), resulting in a total of 2150 first-time PWID at baseline in the Remand Prison Project included in the data analysis.

### Determinant exposures and injection risk behaviour outcomes

The current analysis used the following three injection risk behaviours as outcomes based on self-reported answers within a 12-month recall period: (1) having shared injection drug solution with somebody (yes or no); (2) having lent out already used injection equipment to somebody (yes or no); and (3) having received already used injection equipment from somebody (yes or no).

Based on previous research [22], the following 10 determinants were selected for inclusion in the statistical analysis: gender (woman or man), place of birth (Sweden, Europe (WHO region), or the rest of the world), self-reported living situation (homeless, living with somebody, or having a housing contract), and number of times previously in prison (0, 1–2, or 3 or more). The division into age groups follows the age-related pattern of the Swedish school system [23], which included self-reported age when starting drugs (≤ 13, 14–16, 17–19, 20–24, or 25 years or older) and self-reported age when starting injection drugs (≤ 16, 17–19, 20–24, 25–29, or 30 years or older). Furthermore, we evaluated the self-reported type of drug used when starting drug injection (amphetamine, heroin, or other), including the self-reported most used drug during the last 12 months (injecting amphetamine, heroin, or any other type of drug; cannabis, oral amphetamine, smoked heroin, alcohol, benzodiazepine (non-injection), buprenorphine (non-injection), cocaine, and other drugs that was not injected), time from self-reported start of drug injection to the current interview (≤ 5, 6–10, 11–15, 16–20, 21–25, 26–30, 31–35, or 36+ years) and the calendar year for the interview.

### Statistical analysis

Descriptive analyses were performed to describe socio-environmental characteristics for the study population, and categorical data were described as percentages. Data on the living situation and having shared injection drug solution was missing for 2002. Multivariable logistic models were used to study the association between the three injection risk behaviour outcomes and each of the 10 potential determinants described above. The relationship between the three outcomes and year of interview was modelled assuming linearity. Sensitivity analyses were conducted, including a quadratic form of the year of examination and polynomial b-spline with 3 degrees of freedom, and there were no significant differences in the estimates. Nonetheless, the model with polynomial b-splines for the variable year of examination was used to model the predicted values for the three injection risk behaviour outcomes. All putative variables were kept in the final model. Confidence intervals (CI) were set as 95%, and *p* < 0.05 was considered statistically significant. Stata 13 was used for the analysis.

## Results

**Fig. 1:**
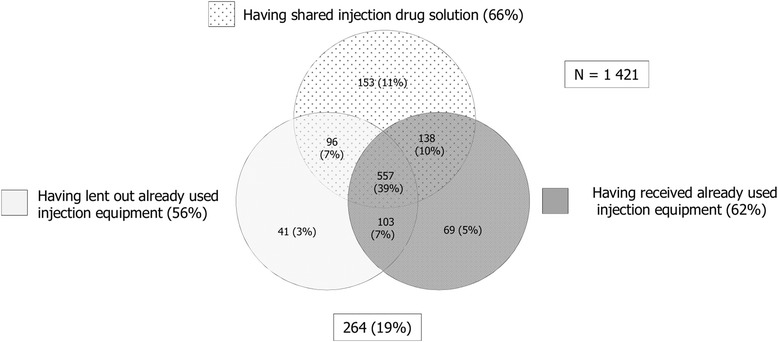
Respondent distribution per injection risk behaviour outcome

Of the 2150 respondents who were included in the final analysis, 84% were men and 68% were born in Sweden. The median age was 37 years (interquartile range of 16 years) for male versus 35 years (interquartile range of 17 years) for female participants. The majority (78%) had used non-injection drugs before age 17, and most reported cannabis as their first drug (79%). More than half (53%) had injected drugs before age 20, and 72% said that amphetamine was the first injection drug they had used, while 25% had used heroin on their first injection. When asked about drug preferences during the last 12 months, 65% said they preferred an injectable drug over a non-injectable, two-thirds (65%) predominantly injected amphetamine while one-third injected heroin (33%). Cannabis was dominant among the 35% who said they preferred to use non-injection drugs while still sometimes injecting drugs. Almost one third (30%) of all interviewees reported they had been homeless prior to the current arrest, and 72% had been to a remand prison at an earlier occasion (but never participated in the Swedish Remand Prison Project before). Approximately two thirds (66%) reported they had shared injection drug solution the last year. Similarly, 56% acknowledged that they had lent out used needles or syringes to others, and 62% stated that they had received already used injection equipment from somebody during the last 12 months (Table 1). Thirty-nine percent reported they had engaged in all three injection risk behaviours over the last 12 months (Fig. 1).

**Table 1:** Determinant characteristics for the study population per injection risk behaviour, (2002–2012, *N* = 2150)

	Having shared injection drug solution		Having lent out already used injection equipment		Having received already used injection equipment	
	Yes (*N*, %)	Total (*N* = 100%)	Yes (*N*, %)	Total (*N* = 100%)	Yes (*N*, %)	Total (*N* = 100%)
Gender						
Woman	185 (76.8)	241	195 (62.9)	310	201 (65)	309
Man	773 (63.9)	1210	835 (54.2)	1541	942 (61)	1543
Total	958 (66)	1451	1030 (55.6)	1851	1143 (61.7)	1852
Place of birth						
Sweden	673 (67.8)	992	723 (57.2)	1265	785 (62)	1266
Europe (excl. Sweden)	193 (63.1)	306	213 (52.7)	404	248 (61.2)	405
Rest of the world	76 (57.6)	132	78 (49.1)	159	97 (61.4)	158
Total	942 (65.9)	1430	1014 (55.5)	1828	1130 (61.8)	1829
Living situation (2003–2012)						
Homeless	327 (72)	454	276 (60.7)	455	312 (68.3)	457
Living with somebody	429 (65)	660	371 (55.5)	668	419 (62.8)	667
Own housing contract	192 (59.8)	321	148 (46.4)	319	144 (45.3)	318
Total	948 (66.1)	1435	795 (55.1)	1442	875 (60.7)	1442
Number of times in prison						
0	254 (66.5)	382	237 (57.9)	409	277 (67.4)	411
1–2	243 (64.6)	376	233 (55)	424	253 (59.7)	424
3 or more	450 (67.2)	670	432 (54.7)	790	462 (58.6)	788
Total	947 (66.3)	1428	902 (55.6)	1623	992 (61.1)	1623
Age when starting drugs						
≤ 13 years	399 (69.5)	574	427 (58.8)	726	484 (66.5)	728
14–16 years	331 (65.4)	506	358 (55.7)	643	399 (62.2)	641
17–19 years	99 (62.3)	159	105 (53)	198	111 (56.1)	198
20–24 years	43 (58.9)	73	52 (50.5)	103	56 (54.4)	103
≥ 25 years	29 (58)	50	24 (39.3)	61	24 (39.3)	61
Total	901 (66.2)	1362	966 (55.8)	1731	1074 (62)	1731
Age when starting injection drugs						
≤ 16 years	270 (68.7)	393	301 (58)	519	334 (64.5)	518
17–19 years	254 (70.6)	360	265 (58)	453	273 (60.3)	453
20–24 years	226 (64.4)	351	239 (53.6)	446	288 (64.3)	448
25–29 years	92 (61.7)	149	107 (53.5)	200	117 (57.9)	202
≥ 30 years	111 (61.3)	181	112 (51.6)	217	125 (57.9)	216
Total	953 (66.5)	1434	1024 (55.8)	1835	1137 (61.9)	1837
Type of drug at injection debut						
Amphetamine	707 (68.8)	1027	723 (54.8)	1320	786 (59.5)	1320
Heroin	221 (61.7)	358	269 (58.4)	461	319 (68.8)	464
Other	20 (48.8)	41	25 (55.6)	45	27 (61.4)	44
Total	948 (66.5)	1426	1017 (55.7)	1826	1132 (61.9)	1828
Most used drug the last 12 months						
Amphetamine (inject)	528 (79.5)	664	537 (61.5)	873	561 (64.4)	871
Heroin (inject)	216 (66.5)	325	278 (62.6)	444	319 (71.8)	444
Other (inject)	12 (46.2)	26	14 (48.3)	29	18 (62.1)	29
Total	756 (74.5)	1015	829 (61.6)	1346	898 (66.8)	1344
Cannabis	59 (52.2)	113	55 (40.1)	137	63 (46)	137
Amphetamine (oral)	22 (55)	40	14 (34.1)	41	12 (29.3)	41
Heroine (smoke)	5 (45.5)	11	5 (38.5)	13	6 (46.2)	13
Alcohol	46 (41.8)	110	47 (35.3)	133	68 (50.7)	134
Benzodiazepine (oral)	32 (50)	64	38 (52.8)	72	42 (57.5)	73
Buprenorphine (oral)	20 (47.6)	42	18 (40.9)	44	30 (66.7)	45
Cocaine (sniffing)	2 (11.8)	17	4 (23.5)	17	5 (29.4)	17
Other (non-injectable drugs)	11 (37.9)	29	16 (44.4)	36	16 (44.4)	36
Total	197 (46.2)	426	197 (40)	493	242 (48.8)	496
Time from injection drug debut to current interview						
≤ 5 years	229 (63.8)	359	251 (56.5)	444	292 (65.8)	444
6–10 years	195 (68.2)	286	208 (59.1)	352	250 (70.6)	354
11–15 years	122 (68.5)	178	143 (61.4)	233	155 (66.2)	234
16–20 years	94 (63.1)	149	113 (56.2)	201	119 (59.2)	201
21–25 years	101 (70.6)	143	113 (55.1)	205	125 (61)	205
26–30 years	87 (69)	126	86 (54.1)	159	90 (56.6)	159
31–35 years	68 (66)	103	65 (49.2)	132	69 (52.3)	132
≥ 36 years	58 (63.7)	91	45 (40.9)	110	38 (34.9)	109
Total	954 (66.5)	1435	1024 (55.8)	1836	1138 (61.9)	1838

### Socio-environmental determinants

Women reported a significantly higher prevalence of injection risk behaviour than men; 77% of the women vs 64% of the men had shared injection drug solution, 63 vs 54% had lent out injection equipment, and 65 vs 61% had received already used injection equipment over the last 12 months (Table 1). When adjusting for confounders (Table 2), women were found to be 51% more likely than men to share injection drug solution (OR 1.51, 95% CI 1.03; 2.21). With regard to country of birth, 68% were Swedish-born, and 22% were from other parts of Europe (data not shown). PWID born outside of Europe were 32% less likely (although not statistically significant) (OR 0.68, 95% CI 0.44; 1.04) compared to Swedish-born to have lent out used injection equipment. Homeless PWID were much more likely to report risky injection behaviours than those with a more stable living situation. Having a housing contract was associated with a 37% lower risk of sharing injection drug solution (OR 0.63, 95% CI 0.44; 0.90), a 43% lower risk of lending out already used injection equipment (OR 0.57, 95% CI 0.41; 0.80), and a 59% lower risk of receiving already used injection equipment (OR 0.41, 95% CI 0.29; 0.58).

**Table 2:** Outcome adjusted odds ratios per injection risk behaviour, (2002–2012, *N* = 2150)

	Having shared injection drug solution	*P* value	Having lent out already used injection equipment	*P* value	Having received already used injection equipment	*P* value
Gender						
Man	1		1		1	
Woman	1.51 (1.03; 2.21)	0.036	1.31 (0.94; 1.83)	0.113	0.95 (0.67; 1.34)	0.755
Place of birth						
Sweden	1		1	1		
Europe (excl. Sweden)	0.96 (0.69; 1.33)	0.794	0.89 (0.66; 1.20)	0.45	0.88 (0.64; 1.21)	0.43
Rest of the world	0.9 (0.57; 1.42)	0.644	0.68 (0.44; 1.04)	0.077	0.87 (0.56; 1.38)	0.563
Living situation						
Homeless	1		1		1	
Living with somebody	0.7 (0.52; 0.96)	0.027	0.8 (0.61; 1.06)	0.121	0.71 (0.53; 0.95)	0.022
Own housing contract	0.63 (0.44; 0.9)	0.012	0.57 (0.41; 0.80)	0.001	0.41 (0.29; 0.58)	< 0.001
Number of times in prison						
0	1		1		1	
1–2	1 (0.69; 1.43)	0.986	0.94 (0.67; 1.3)	0.695	0.75 (0.53; 1.05)	0.097
3 or more	1 (0.69; 1.45)	0.994	0.91 (0.65; 1.27)	0.567	0.8 (0.56; 1.13)	0.205
Age when starting drugs						
17–19 years	1		1		1	
≤ 13 years	1.24 (0.79; 1.93)	0.347	1.23 (0.82; 1.85)	0.313	1.48 (0.97; 2.26)	0.067
14–16 years	0.97 (0.64; 1.48)	0.891	1.12 (0.76; 1.65)	0.558	1.15 (0.77; 1.71)	0.507
20–24 years	0.93 (0.49; 1.77)	0.815	0.73 (0.40; 1.36)	0.322	0.87 (0.46; 1.64)	0.666
≥ 25 years	0.95 (0.43; 2.07)	0.889	0.83 (0.40; 1.72)	0.615	0.42 (0.20; 0.89)	0.024
Age when starting injection drugs						
17–19 years	1		1		1	
≤ 16 years	0.88 (0.59; 1.31)	0.53	1 (0.7; 1.42)	0.989	1.43 (0.99; 2.08)	0.06
20–24 years	0.85 (0.59; 1.24)	0.407	0.76 (0.54; 1.06)	0.103	1.07 (0.75; 1.52)	0.706
25–29 years	0.65 (0.4; 1.06)	0.083	0.78 (0.5; 1.22)	0.28	0.96 (0.6; 1.54)	0.873
≥ 30 years	0.46 (0.29; 0.76)	0.002	0.67 (0.43; 1.05)	0.078	0.8 (0.5; 1.28)	0.35
Type of drug at injection debut						
Heroin	1		1		1	
Amphetamine	0.98 (0.69; 1.39)	0.91	0.87 (0.63; 1.21)	0.413	0.8 (0.57; 1.14)	0.217
Other	0.73 (0.33; 1.59)	0.426	1.27 (0.58; 2.74)	0.551	1.22 (0.53; 2.8)	0.635
Most used drug the last 12 months						
Heroin (inject)	1		1		1	
Amphetamine (inject)	2.43 (1.64; 3.62)	< 0.001	0.95 (0.67; 1.37)	0.80	0.88 (0.6; 1.29)	0.525
Other (inject)	0.49 (0.18; 1.3)	0.152	0.40 (0.15; 1.09)	0.072	0.43 (0.15; 1.21)	0.109
Cannabis	0.54 (0.33; 0.9)	0.017	0.38 (0.23; 0.63)	< 0.001	0.34 (0.21; 0.57)	< 0.001
Amphetamine (oral)	0.73 (0.33; 1.6)	0.427	0.34 (0.15; 0.79)	0.012	0.2 (0.08; 0.5)	0.001
Heroine (smoke)	0.39 (0.11; 1.37)	0.141	0.29 (0.08; 1.02)	0.053	0.23 (0.07; 0.77)	0.018
Alcohol	0.37 (0.22; 0.62)	< 0.001	0.28 (0.16; 0.47)	< 0.001	0.43 (0.25; 0.73)	0.002
Benzodiazepine (oral)	0.44 (0.24; 0.81)	0.008	0.43 (0.24; 0.79)	0.007	0.48 (0.26; 0.9)	0.021
Buprenorphine (oral)	0.46 (0.23; 0.96)	0.037	0.31 (0.15; 0.66)	0.002	0.78 (0.37; 1.66)	0.517
Cocaine sniffing)	0.09 (0.02; 0.45)	0.003	0.18 (0.05; 0.71)	0.014	0.16 (0.05; 0.58)	0.005
Other (non-injectable drugs)	0.36 (0.16; 0.85)	0.02	0.39 (0.17; 0.9)	0.027	0.22 (0.09; 0.53)	0.001
Time from injection drug debut to current interview						
≤ 5 years	1		1		1	
6–10 years	1.01 (0.69; 1.5)	0.941	0.89 (0.62; 1.28)	0.529	0.91 (0.62; 1.33)	0.618
11–15 years	1.11 (0.69; 1.79)	0.661	0.96 (0.62; 1.49)	0.87	0.8 (0.51; 1.27)	0.347
16–20 years	0.7 (0.42; 1.16)	0.163	0.68 (0.43; 1.09)	0.107	0.53 (0.33; 0.86)	0.01
21–25 years	0.91 (0.53; 1.57)	0.741	0.87 (0.53; 1.4)	0.558	0.68 (0.41; 1.12)	0.13
26–30 years	0.67 (0.36; 1.22)	0.19	0.58 (0.34; 1)	0.051	0.34 (0.2; 0.6)	< 0.001
31–35 years	0.52 (0.28; 0.98)	0.044	0.58 (0.33; 1.04)	0.069	0.47 (0.26; 0.85)	0.013
≥ 36 years	0.72 (0.34; 1.51)	0.388	0.4 (0.2; 0.77)	0.006	0.2 (0.1; 0.4)	< 0.001
Calendar year of interview						
	0.87 (0.83; 0.92)	< 0.001	0.94 (0.9; 0.99)	0.022	0.89 (0.85; 0.94)	< 0.001

### Drug-related determinants

A young age when starting drugs was strongly associated with all three injection risk behaviour outcomes. Individuals who began injecting as adults, i.e. at age 25–29 or 30 years or older, were 35% although not statistically significant (n.s.) and 54% less likely to share drug solutions (OR 0.65, 95% CI 0.40; 1.06, vs OR 0.46, 95% CI 0.29; 0.76) compared to those who started to inject drugs before age 20. Similarly, those who started to inject drugs after age 30 had a 33% (n.s.) lower risk of having lent out used injection equipment (OR 0.67, 95% CI 0.43; 1.05). The association between a young drug debut age and injection risk behaviour was stronger, when comparing those who reported that they had begun to use drugs before age 14. These individuals had a 48% (n.s.) higher risk (OR 1.48, 95% CI 0.97; 2.26) of receiving used injection equipment compared to those who were slightly older when they started to use drugs, i.e. 17–19 years of age. Similarly, those who started using injection drugs before age 17 had a 43% higher risk (borderline significant, OR 1.43, 95% CI 0.99; 2.08) for having received used injection equipment compared to those who started between 17 and 19 years of age. Those who mainly had injected amphetamine over the past 12 months were more than twice as likely (OR 2.43, 95% CI 1.64; 3.62) to have shared injection drug solutions compared to those who injected heroin. In a sub-analysis of PWID reporting mainly using a non-injectable drug (*N* = 223, data not shown), we found that cocaine users were at a lower risk than cannabis users (OR 0.17, 95% CI 0.04; 0.85) to have shared injection drug solution, and those who used oral buprenorphine were more than twice as likely (borderline significant OR 2.27, 95% CI 0.99; 5.21) to receive used injection equipment.

**Fig. 2:**
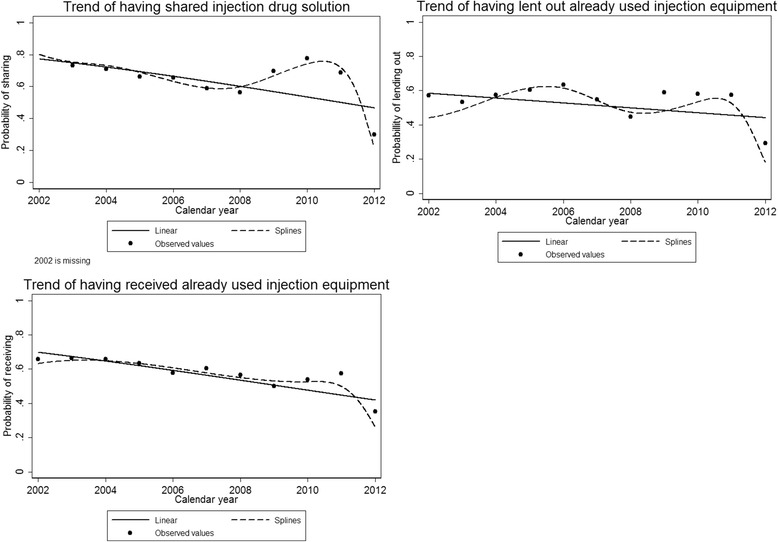
Observed and predicted values of the probability of three key injection risk behaviours between 2002 and 2012

Even after adjusting for gender, place of birth living situation, number of prior times in prison, age at drug debut, age when starting to use injection drugs, type of drug used when starting injection drugs, most commonly used drug during the last 12 months, and time from start of using injection drugs to time of interview, we found a strong effect of calendar time. For each calendar year observed during the study period (2002–2012), there was a decrease in the odds of shared injection drug solutions (OR 0.87, 95% CI 0.83; 0.92) and for having lent out (OR 0.94, 95% CI 0.9; 0.99) or received (OR 0.89, 95% CI 0.85; 0.94) already used injection equipment (Table 2 and Fig. 2).

## Discussion

PWID in Sweden are hard to reach and heavily affected by HCV and HIV infection due to a high prevalence of injection risk behaviours despite access to OST as well as a recent expansion of NEPs over the last years. Remand prisons in Sweden constitute a suitable platform for identifying this hard-to-reach group and for conducting sentinel surveillance for blood-borne infections and risk behaviours among PWID. We analysed socio-demographic and drug-related determinants for three key injection risk behaviours among PWID in Swedish remand prisons during 2002–2012. Between 56 and 66% of the respondents reported to have engaged in any of the three injection risk behaviours already at entry into the project. We found that female PWID were significantly more likely than their male peers to share injection drug solutions. Research has previously shown a higher risk for women to share needles [24–26] and ancillary equipment [25]. A young age at both non-injection debut as well as when starting to use injection drugs was also found to be strongly associated with injection risk behaviour. Those who began using drugs at a very young age, before age 14, were the ones with the most hazardous injection risk behaviour, emphasising the importance of very early prevention interventions against drug use. Previous studies from Karachi [27], China [28], and Ukraine [29] have also found that young people and female PWID [25] are most vulnerable and at highest risk to engage in unsafe injection behaviour. Therefore, we believe that early prevention and harm reduction services should expand and improve their efforts to target young people, and for those who already inject, tailor-make programs, specifically NEPs in the Swedish case, to attract more female PWID.

Our study specifically used age categories related to the mandatory school system in Sweden given that children are often re-located to a new school and geographical setting at two critical points in life, at age 13 (moving from primary school to intermediary school) and at age 17 (when they have an option to continue to upper secondary school or leave). Almost all PWID began to use drugs when they were still in mandatory school at the intermediary level, suggesting that the school arena is an important opportunity to reach young people at risk of starting to use drugs, or those who have already had their injection debut. Up until 2017, Swedish law prohibited needle exchange among people younger than 20 years, an age-threshold that has posed a great challenge for effective prevention of HCV and HIV among young PWID [19]. In March 2017, the parliament passed a new NEP law which allows those aged 18 years or older to exchange injection equipment [30]. However, almost half of PWID in our study population (53%) started to inject drugs before age 18 (the median age of injection drug debut was 19), and those who started injecting drugs at a very early age were those most at risk of using non-sterile equipment. In Stockholm, for example, where the first NEP opened in 2013, previous HIV outbreaks in PWID have been associated with no or limited access to NEPs [12]. Furthermore, studies from the same region have shown that as much as 50% of PWID have HCV antibodies 2 years after starting to use injection drugs [15]. Additionally, knowing a person’s HCV status was not sufficient to prevent sharing of injection equipment [31]. Our findings are supported by data from Estonia, where people who started using injection drugs at an early age doubled their risk of becoming HIV positive [32]. The new NEP law will improve conditions for PWID in Sweden, but it will not help the young and most at risk who start to inject drugs before age 18. Those who have their drug debut at a later age can be associated with a more stable life situation, including employment, education and a larger social network. However, older individuals may also be more mature and have a higher level of self-control, as indicated by a study in Atlanta, USA, which concluded that it was possible to maintain a normal social role in society as long as one maintained self-control in terms of drug use [33].

In our study, those who mainly injected heroin were less likely to share injection drug solution compared to those who preferred to inject amphetamine. Similar results have been found in Georgia, where ephedrine users were more likely to engage in unsafe injection behaviour compared to heroin users [34], and in Ontario, Canada, where amphetamine users were more prone to share injection equipment [35]. This difference in sharing patterns between those who inject heroin vs amphetamine is important to consider when designing prevention and harm reduction activities for HCV in particular since sharing of paraphernalia (injection drug solution) alone is a strong risk factor for HCV infection [36]. We also found a surprisingly strong prevalence of unsafe injection risk behaviour among those who most often used non-injectable drugs. Oral buprenorphine users were more than twice as likely to use unsterile injection equipment as those who reported oral cannabis as their most frequently used drug. These results demonstrate the importance for harm reduction services to target general drug use while specifically tailoring interventions to those PWID who report illicit use of buprenorphine. One study in Providence, USA, found that a majority of buprenorphine users used this drug for self-medication purposes [37]. This observation could indicate the existence of a sub-population with an assumed higher level of motivation to control or willingness to stop using drugs in our study population, who are either enrolled in an OST programme or on self-imposed medication. Not surprisingly, unstable living and housing circumstances, especially being homeless, was a strong determinant for all three injection risk behaviours. Homelessness has previously been shown to be a risk factor for both HIV [38] and HCV infection among PWID [39] as well as for sharing paraphernalia [35] and having a more accepting attitude towards sharing [40]. Unstable housing conditions have also acted as a barrier for both HIV [41] and HCV treatment [42] among PWID. Therefore, improved access to stable housing conditions for PWID may reduce their risky injection behaviour.

Previous prison experience has been shown to lead to higher levels of injection risk behaviour [43]; but results were inconclusive on this topic in our analysis, possibly explained by the Swedish prison system environment, i.e. access to general health care, voluntary counselling and testing, infectious disease treatment, OST and possibly a more rigorous control of the availability of syringes, needles, paraphernalia and drugs in the prison environment, compared to countries that either have NEPs in prison or where access to drugs may be easier. However, we found that the longer a person managed to stay out of the prison environment (here measured as time from starting to use injection drugs to the time (calendar year) of the baseline project interview), the lower the injection risk behaviour. This is plausible given that the longer somebody can avoid to end up in a remand prison, the more likely it is that he or she manages to exert some level of control over his or her drug use.

A clear decreasing trend for all three injection risk behaviour outcomes was shown over the last decade (2002–2012). This finding could be explained by several events on both the national and regional level. In 2006, a law was passed in Sweden that allowed county councils to start NEPs [44]; in that same year, the Swedish government launched a national strategy, including funding for HIV preventive work specifically targeting PWID [45]. In 2007–2008, an HIV outbreak among PWID in Stockholm County (at the time without any NEP) increased the level of testing on the group, including specific information, education and communication interventions [12, 46]. In 2009, the National Board of Health and Welfare changed its recommendation on OST, easing the restrictions for participation in the programme and allowing more people to enrol. In addition, the Social Remand Prison Project maintained high levels of counselling, testing and vaccination among PWID, reaching many people who use drugs. All the above-mentioned factors may have contributed to the observed general and overall time-related decline in injection risk behaviour among PWID (calendar year, Fig. 2).

## Conclusions

Our results show that being a woman, starting to use drugs and/or injecting drugs at an early age, injecting amphetamine and using certain non-injection drugs, being homeless and ending up early on in prison were all significant determinants for having an increased level of injection risk behaviour. Time was an important protective determinant probably related to an expansion of harm reduction initiatives. Different prevention efforts work in parallel at different levels in society, and policymakers and decision-makers should ensure that all PWID in need can access harm reduction services regardless of age and existing services must be viewed as user-friendly and accessible to both men and women, young and old.

### Limitations

This study was conducted in a remand prison, a confined environment where PWID were in custody pending a trial or possible release, which is not entirely comparable with PWID in the general community. Respondents were identified among newly arrested PWID, and the great majority were held in custody for less than 24 h. The interview related to their behaviour focused on the time preceding the arrest. It is unlikely that the time spent in remand prison itself influenced the answers, and we do not think any influence of drugs significantly affected the answers because the interviewers always waited until the respondents were sufficiently sober to properly understand the information provided and give adequate informed consent. Self-reported retrospective behaviour during the last 12 months could be subject to recall bias or be biased by an aversion to respond to sensitive questions. However, the interviewers reported an overall impression that most participants enjoyed the interviews, which offered a break in otherwise tedious waiting for a pending trial hearing. Another potential source of bias was the small number of young and female PWID, but the sample represents the proportion of young people and women who are arrested and appear in the remand prison setting.

### Strengths

The study strengths include the large number of participants who were prospectively enrolled and the complete data set available for data analysis. The remand prison setting is a transitional environment where PWID that are most likely to be arrested often transit through, meaning, our data provide a reasonably good reflection of the risk situation experienced by PWID in the general community, and the high response rate increases the generalisability of our sample.
